# Prevalence of occupational exposure to HIV and utilization of HIV post-exposure prophylaxis among health staff at Bule Hora General Hospital, Bule Hora, Ethiopia

**DOI:** 10.11604/pamj.2020.37.333.25680

**Published:** 2020-12-10

**Authors:** Girish Degavi, Shiferaw Gelchu Adola, Hazaratali Panari, Shivaji Pawar, Chala Wata Dereso

**Affiliations:** 1 Department of Nursing, College of Health and Medical Science, Bule Hora University, Hagere Maryam, Ethiopia,; 2 Department of Nursing, Institute of Medicine and Health Sciences, Debre Berhan University, Debre Berhan, Ethiopia,; 3 Krishna Institute of Medical Sciences University, Krishna Institute of Nursing Sciences, Karad, Maharashtra, India,; 4 Bule Hora University, Hagere Maryam, Ethiopia

**Keywords:** Occupational exposure, HIV, Bule Hora Hospital, nurses

## Abstract

**Introduction:**

occupational risk of HIV and low utilization of post-exposure prophylaxis (PEP) among nurses has become a global public health concern. According to the International Labor Organization estimates, 2.02 million people die each year from work-related accidents or diseases. More than 317 million people suffer, and there are an estimated 337 million fatal and non-fatal work-related accidents per year. WHO report indicates, more than 59 million health care workers around the world are exposed to biological hazards and about 10% of HIV among health workers is the result of needle stick injury. This study focused on assessing the prevalence of occupational exposure to HIV post-exposure prophylaxis among nurses at Bule Hora Hospital.

**Methods:**

institutional based cross-sectional study design was conducted from March 2019 to April 2019. In this study, 306 study participants were involved in the study data was collected using a structured and semi-structured questionnaire. The cleaned data (edited) was entered into Epi-Data version 4.4.3.1 and exported to SPSS Statistics Version 20 for analysis.

**Results:**

high incidence (61.6%) of occupational exposure to HIV was found in this study. The two leading types of exposure were blood splash 40.5% and needle stick 37.8% injury followed by giving injection 27% and uncooperativeness 21.6% subsequently. About 35.1% of nurses were found to be not using personal protective equipment’s while being exposed to HIV infection while on work.

**Conclusion:**

occupational exposure to HIV is quite common among nurses in Bule Hora Hospital. Blood splash exposure and exposure to needle stick injury are believed to be the commonest types of workplace risks. Nearly 29.7% of nurses have no training on infection prevention and management while at work. Nearly 50% of the exposed nurses didn’t report the incident to the concerned authorities. One fourth of the sources of exposure were unscreened and among the screened sources of exposure 42.9% were found to be HIV positive.

## Introduction

Nurses encounter various work-related risks in the form of injuries or infection due to the nature of the workplace and their obligations towards their job that put them in the forefront of occupational related hazards [1]. Medical doctors and nurses are considered at most risk group of needle-stick injuries as compared to other health service workers [2]. Accordingly, they are handily presented with transferable illnesses including hazardous blood borne microorganisms which are transmitted by means of needle stick injury, blood, and body liquid splash. Study directed in Iran shows that the prevalence of exposure to workplace risk among nursing and maternity specialists were 50.7% and another data revealed in Serbia uncovered that the highest prevalence 68.6% of work-related accidents was among medical caretakers [3, 4].

In sub-Saharan Africa, the prevalence of occupational exposure to HIV is also high. Study conducted in Tanzania showed that 50.6% health care workers participated in the study experienced harmful occupational exposure [5]. In Kenya 50% of nurses reported that they were exposed to percutaneous injuries [6]. Study´s conducted in Ethiopia revealed that the overall prevalence of blood/body fluid exposure and sharp injury was 78.3% [7]. Among sub-Saharan Africa, the prevalence of occupational exposure is higher in Ethiopia, where the prevalence of HIV infection is also very high [8, 9]. The use of HIV PEP brings significant efficacy by preventing HIV sero-conversion. Studies done on animals shows risk of sero-conversion among animals exposed to PEP were lowered by 89% [10]. Immediate use of HIV post-exposure prophylaxis is believed to reduce occurrence HIV infection by 80% [11]. Despite an increased occupational hazard in the health care settings, there is improper practice guidelines and motives among the health care force to protect themselves from HIV risk exposure evidenced by negligent utilization of HIV post-exposure prophylaxis among nurses [12]. In general, occupational exposure is becoming a serious problem, but it was not getting adequate focus and needed concern. Therefore, this cross-sectional study was conducted to assess the prevalence of occupational exposure and determine the magnitude of utilization of PEP among health care staff including nurses at Bule Hora General Hospital.

## Methods

An institution based cross-sectional study design was incorporated on 306 staffs at Bule Hora General Hospital which is located in Bule Hora town, which is the capital city of West Guji Zone. Bule Hora General Hospital (BHH) is the largest hospital of all hospitals included in this study area. These hospitals were selected due to its accessibility for the present study.

Staff nurses and doctors working in Bule Hora Hospital were included in the study. Through literature search the prevalence studied at Jima public hospital (78.3%) were taken as reference [7]. Using a 78.3% as prevalence rate, incorporating precision value of 0.05 and a Z-value of 1.96, the formula yielded a minimum sample size of 261 adding (10%) of null respondents, the final sample size was decided to be 306.

The study participants were selected using systematic random sampling using first ‘k^th^’ value in the overall health staff list of the study area obtained from local public health office. Health staff who were giving direct care to the patients were the study participants of whom (26%) were medical doctors including 7 specialists, (64%) general nursing staff and (10%) midwifery nursing staff. All participants possessed with different levels of qualification in their respective field. The dependent variables included in the study were exposure to occupational hazards related to HIV and utilization of PEP. The independent variables were socio-demographic factors such as age, sex, educational status, marital status, work experience, and position/title in hospital. Organizational factors, Nurse and patient behavioral factors were studied on use of personal protective equipment, Organization and nurse related factors including stigma/discrimination, fear of drug side effects, awareness on HIV PEP, availability of PEP drugs, and availability of HIV PEP protocol. Patient factors include HIV status.

The data were collected from March 2019 to April 2019 for 2 consecutive months. The assessment was done using pretested, semi structured administered questionnaires. The questionnaire had three part one: Sociodemographic characteristics of the nurses, part two: Prevalence of occupational exposure and part three: Utilization of HIV-PEP medications. Prior to the actual data collection, pre-testing was done on 5% of the total study participants at Kerch Hospital in West Guji Zone, Ethiopia. which were not included in the actual study and based on the findings necessary, amendment was made regarding its consistency, clarity, and logical adequacy. The data was coded, checked for error, missing value must dealt with and cleaned data (edited) will be entered into Epi-Data version 4.4.3.1 and exported to SPSS statistics version 20 for analysis.

The statistical analysis focused on quantity and quality, summarizing, analyzing and assessing and bringing out conclusions of the collected data. The results of descriptive statistics were summarized and presented by tables, charts, and graphs. Percentage frequency and mean was been calculated. To enable participants to disclose and respond openly, 4 BSc nurses for data collection and 2 MSc nurses for supervision of data collection were recruited in this study. The study assistants were trained for one day intensively on the study instrument and data collection procedure that includes the relevance of the study, objective of the study, confidentiality of the information, and informed consent.

### Operational definition

**Occupational exposure to HIV:** doctors and Nurses who were exposed to needle stick cuts, blood or other body fluid splashes occurring at the workplace during performing procedures.

**Post-exposure prophylaxis use:** is the timely provision of anti-retroviral (ARV) medication following exposure to potentially infected blood or other body fluids to minimize the risk of acquiring infection; The drugs should be provided within 72 hours, daily for 28 days; recommended drugs for low-risk HIV exposure are a combination of Tenofovir (TDF) + Lamivudine (3TC) or Zidovudine (AZT) + Lamivudine (3TC) while for high risk exposures triple therapy should be used i.e. Tenofovir (TDF)/Zidovudine (AZT) + Lamivudine (3TC) and Efavirenz (EFV).

**Ethical approval:** ethical clearance was obtained from Bule Hora University, College of Health and Medical Science, an institutional review board (BHU-IRB) of the research committee. Respondents were informed about the purpose and objective of the study. The information was collected after obtaining verbal consent from each participant. Verbal consent was wanted from all the informed respondents before the start of each interview. Respondents were allowed to refuse or discontinue or participation at any time they want. Information was recorded anonymously and confidentiality and beneficence were assured throughout the study.

## Results

**Socio-demographic characteristics of participants:** the study was conducted among 306 staffs with a response rate of 97%. The response rate is 100%. Majority (60%) of participants were female. The age of the study participants ranged from 22 to 57 with 28 median ages. About 57% were single in marital status and all most all 93% were staff nurses. In addition, 63.3% were Diploma holders. Regarding the departments they are working, 15%, 20%, and 18.3% of nurses were working in outpatient department, emergency, and medical wards, respectively (Table 1).

**Table 1:** socio-demographic characteristic of participants, Bule Hora Hospital, 2020

Variable	Response	Frequency (N)	Percent (%)
Sex	Male	122	40
	Female	183	60
Age	≤ 30	229	75
	31-40	56	18
	>40	20	7
Marital status	Single	173	57
	Married	132	43
Title in the hospital	Staff nurse	285	93
	Head nurse	20	7
Education level	Diploma	194	63.3
	Bsc degree	97	31.7
	Master degree	15	5
Working ward	Pediatric ward	420	6.7
	Medical-ward	56	18.3
	Gynecology ward	15	5
	Surgical ward	41	13.3
	Operating theatre	35	11.7
	Outpatient department	15	25
	Emergency ward	61	20
Service year	≤5 years	204	66.3
	>5 years	102	33.7

* Significant association at p < 0.05

**Prevalence of occupational exposure to HIV among nurses at BHH, 2020:** the prevalence of occupational exposure to HIV among the 306 study participants is 37 (61.6%). About 40.5%, 37.8%, 13.5%, and 8.1% nurses experienced blood splash, needle sticks, had more than two exposures, and mucus slash, respectively. Giving the injection 27% and uncooperativeness of patients 21.6% were among the common activities that exposed nurses to occupational exposure to HIV. Majority (46%) of nurses had experienced one-time exposure to HIV in the past 12 months. Non availability of equipments and negligence were the reasons for not using personal protective equipment; 70.3% of nurses have taken training on infection prevention and more than half (62.2%) of the injuries occurred at daytime. Most sources of exposures were screened (73.7%), and 42.9% were HIV positive (Table 2).

**Table 2:** prevalence of occupational exposure to HIV among health staff at BHH, 2020 (n = 306)

Variable	Response	Percent (%)
Exposed activities	Giving injections	27
	Recapping needles	5.4
	During surgery	13.5
	Sudden movement of the patient	21.6
	Collection of wastes	10.9
	≥2 activities	21.6
Time of last exposures experienced	Within 3 months	21.6
	Within 6 months	27
	In the past 12 month	46
	Greater than a year	5.4
Use of personal protective equipment at time of exposure	Yes	59.5
	No	35.1
	I don’t remember	5.4
Reason for not using personal protective equipment	Equipment not available	54.5
	Negligence	45.5
Training on infection prevention including PEP	Yes	70.3
	No	29.7
Working shift at exposure time	Day	62.2
	Night	37.8
Total working hour/week	<=40	51.4
	>40	48.6
Reporting the accident	Yes	51.4
	No	48.6
Post-exposure screening of the source of exposure	Yes	73.7
	No	26.3
HIV status of the source of exposure	Positive	42.9
	Negative	57.1
Mean working hours per week	49.2 + 11.15 SD	

* Significant association at p < 0.05

**Utilization of HIV-PEP medications among nurses at BHH:** overall, among 61.6% who had occupational exposure to HIV, 24.3% used HIV-PEP. Of this, 91.9% heard about PEP but more than half (62.2%) have not taken training on PEP. Majority of nurses reported as PEP medication is available and 40.5% of the participants know that PEP services are provided 24 9mm hours and 59.5% do not know the availability of the service at BHH. Of the 42.9% participants exposed to HIV positive sources, 5 nurses did not use PEP immediately (> 2 hours), 3 used PEP immediately and 1 did not use PEP at all. The mean time to initiate the first PEP drug after exposure was 8.88 + 7.97 SD. Among those who started PEP, 77.7% completed. However, 22.3% did not complete PEP medication and the main reason for discontinuation of the PEP was fear of adverse drug effects (Table 3).

**Table 3:** utilization of HIV-PEP medications among health staff at BHH, 2020 (n = 306)

Variables	Response	Percent (%)
Ever used HIV-PEP	Yes	24.3
	No	75.7
Ever heard about HIV-PEP	Yes	91.9
	No	8.1
Ever had training PEP	Yes	37.8
	No	62.2
HIV-PEP medications availability	Yes	75.7
	No	24.3
24 hours HIV-PEP medications administered	Yes	40.5
	No	59.5
Immediately start HIV-PEP medications post-exposure	Yes	33.3
	No	55.6
	Not used PEP at all	11.1
Time lapse from exposure to which PEP was received	Within 2hrs	44.4
	After 2hrs	55.6
Complete your HIV-PEP medications	Yes	77.7
	No	22.3
Reason for discontinuation of the drug	Fear of adverse effect	100
Mean time initiation of HIV PEP	8.88 + 7.97SD	

* Significant association at p < 0.05

## Discussion

This study assessed the prevalence of occupational exposure to HIV post-exposure prophylaxis among nurses at Bule Hora Hospital. This study detected high levels of occupational exposure to HIV, 61.6%. This finding is in line with studies conducted in Serbia (68.6%) [4] and WHO report of 21 African countries 65.7% [13] and lower than similar studies conducted in Jima zone public hospitals and Debre Berhan town in which the prevalence of occupational exposure to HIV were 78.3% [7] and 88.6% [14] respectively. The finding of this study is higher than studies done in Tanzania (50.6%) [5], Kenya (50%) [6], Nigeria (51.0%) [15], Southeast Iran (34.7%) [16], in Gondar town (33.8%) [17] and Hawassa (46%) [18].

In this study, the two leading types of exposure were blood splash 115(40.5%) and needle stick 14(37.8%) injuries. This result is comparable with study in Tanzania which is blood splash 57(47.1) and Needle stick cut 45(37.2%) [5]. But lower than study done in Jima Zone in which body fluid exposure and needle stick injury were 62.6% and 58.8% respectively [7]. According to this study, giving injection 27% and uncooperativeness 21.6% of patients were among the common activities that exposed nurses to occupational exposure to HIV. This finding is in agreement with studies conducted in Hawassa that Emergency situation (28.6%), sudden movement of the patient (23.8%) [18]. In this study, the majority of nurses 17 (46%) had one-time occupational exposure to HIV in their professional performance. this finding is in line with study done in Cameroon that twenty-nine (53.7%) of nurses exposed one time in their working time [12].

In this study, 35.1% nurses did not use personal protective equipment´s during time of exposure. This finding is lower than the study done in North western Tanzania that 45.3% of the participants were not used PPE at the time of exposure [19]. The result of this study showed that non-availability of personal protective equipments (54.5%), reasons for not using was non-availability. This result is lower than the study conducted in Tamil Nadu that the reasons for inappropriate use of PPE was non-availability of PPE 562, 78% [20].

The findings of this study revealed that 70.3% of nurses have taken training on infection prevention. This finding is comparable with a study conducted in Debre Berhan Town, 94(76.4%) nurses were trained on infection prevention [14]. In this study, 51.4% nurses among the exposed reported the accidents to the responsible person. This result is lower than the study done in Tanzania with 68.6% [5] and Uganda 74% [21]. However, higher than the study conducted in Serbia, 40.2% [4]. According to this study, among 61.6% exposures, 24.3% nurses used HIV-PEP. This result is comparable with the study done in Kenya out of 305 reported exposures, only 20% (n=83) took PEP against HIV [6]. However, higher than studies done in Tanzania that 212 reported incidents of NSIs and splash exposure, only 16.7% of exposed HCWs received post-exposure prophylaxis for HIV [19].

This study revealed, most nurses, 91.9% heard about HIV- PEP. This finding was almost similar with a study conducted in Eastern Ethiopia, Hiwotfana hospital that 97.4% of the study participants were aware of HIV PEP [22]. In this study, the majority of nurses 75.7% reported as PEP medication is available and 40.5% of the participants knew that PEP service provided 24hours. This finding in line with similar study conducted in Tanzania that 74.9% reported that the HIV PEP was available at their workplace and more than half 58.1% reported to have a person available to administer the HIV PEP, 24 hours a day [5].

The results of the present study revealed that 33.3% nurses used PEP immediately. This finding is comparable with study done in Eastern Ethiopia, Hiwotfana Hospital that Timely initiation of PEP 26.3% [22] and lower than study conducted in Uganda 58% study participants [21]. The results of this study showed that 77.7% study participants completed PEP among those who started HIV-PEP. This finding is higher than the WHO report of 2018, that 57% of people completed the full course of PEP [8]. As per this study, 22.3% nurses did not complete PEP and the major reason for discontinuation of HIV- PEP among those who used the drug was fear of adverse effects of PEP. The results were in agreement with the study conducted in Mekele town that 19.4% discontinued PEP and 83.3% of those who discontinued were due to adverse effect of the drugs [23], Gaborone 26.6% did not completed PEP, with 71.4% quitting because of adverse drug effects [24]. The reason for the observed difference might be due to the difference in study setting, study population (this study did only among nurses and doctors), study time, and awareness level of study participants regarding the implementation of the universal precaution protocol.

## Conclusion

Occupational exposure to HIV is common among health care professionals. Blood splash exposure and 37.8% nurses had exposure to needle stick injury, common activities that put nurses to HIV exposure. Majority of nurses ignore the importance of infection prevention and post-exposure reporting and necessary screening.

### What is known about this topic

- Even after taking necessary measures by the regulatory and administrative agencies, occupational HIV exposure remains a major health risk among nurses and doctors;
- Needle stick injury and blood splash has become the commonest cause of occupational exposure to HIV among nurses and physicians.

### What this study adds

- Adhering to the utilization of HIV post-exposure prophylaxis is corner stone of prevention of occupational exposure to HIV;
- Appropriate standing orders related to occupational exposure to HIV is needed for health professional’s safety in workplace.
